# Micropulse laser trabeculoplasty on Chinese patients with glaucoma or ocular hypertension: average 35 months follow-up results

**DOI:** 10.1186/s12886-022-02477-w

**Published:** 2022-06-04

**Authors:** Yuting Yang, Xinting Huang, Sheng Liao, Feng Zhang, Jingming Shi, Xuanchu Duan, Ke Liu

**Affiliations:** 1 Department of Ophthalmology, Second Xiangya Hospital, Central South University, Hunan, 410011 China; 2 Department of Ophthalmology & Visual Science, ENT Hospital, Shanghai Medical College, Fudan University, Eye &, Shanghai, 200031 China; 3 Department of Ophthalmology, Third Xiangya Hospital, Central South University, Changsha, Hunan China; 4 Changsha Aier Eye Hospital, Changsha, Hunan China

**Keywords:** Micropulse laser trabeculoplasty, Glaucoma, Ocular hypertension, Laser technology

## Abstract

**Background:**

Glaucoma is a group of eye diseases that can damage the optic nerve and cause vision loss. A novel technology micropulse laser trabeculoplasty (MLT) can use duty-circle subvisible laser pulses to treat glaucoma. The aim of this study is to evaluate the efficacy of 360° MLT to alleviate intraocular pressure (IOP) in patients with glaucoma.

**Methods:**

This was a single-center prospective study on patients treated with 360° MLT using a Diode True-Yellow 577-nm Laser with MicroPulse technology. All the patients were recruited from the Department of Ophthalmology. Follow-up visits were carried out at 1 day, 1 week, 1 month, 3 months, 6 months, 18 months, and 36 months after the procedure. Best-corrected visual acuity (BCVA), Intraocular pressure (IOP), and vertical cup-to-disc ratio (C/D ratio) were measured during the follow-up. Repeated-measures analysis of variance (ANOVA) and Kaplan–Meier analysis were performed to evaluate the outcomes.

**Results:**

A total of 39 eyes from 25 patients were included in this study (10 men/15 women). The average age of patients was 41.47 ± 14.39 years old, and the baseline IOP was 21.13 ± 7.75 mmHg. MLT significantly reduced the IOP at 1 day (range 15.61–19.01, *P* = 0.0218), 3 months (range 16.47–19.22, *P* = 0.0390), and 6 months (range 15.38–18.56, *P* = 0.0332) compared with the baseline. However, by the end of the study, only 21.88% of patients demonstrated a ≥ 20% IOP reduction, while seven eyes needed further laser or surgical treatment. The IOP of glaucoma patients was significantly lower than the ocular hypertension patients at 1 month (*P* = 0.0124), 3 months (*P* = 0.0004), 18 months (*P* = 0.0061) and 36 months (*P* = 0.0119).

**Conclusions:**

Micropulse laser trabeculoplasty reduce IOP in patients with glaucoma or ocular hypertension for a short period, but its lowering efficiency is limited up to 6 months of the follow-up period.

## Background

Glaucoma is the most frequent cause of irreversible blindness, Referred to as a group of eye diseases that can damage the optic nerve and cause vision loss [1]

The managements of glaucoma include anti-glaucoma medications, laser technologies, and filtering and non-filtering surgeries. Laser trabeculoplasty (LTP) has been introduced as a non-invasive procedure to treat multiple types of glaucoma for several years. It applies laser energy to the trabecular meshwork (TM), usually covering 180°-360° range of TM for each treatment [2, 3]. Micropulse laser trabeculoplasty (MLT) is a newly developed technology, first described in 2005. It uses duty-circle subvisible laser pulses and focuses on the anterior TM tissue. Considering its long relaxation time, micropulse laser trabeculoplasty allows the temperature to return to the baseline and avoids heat damage to TM [2]. Several clinical trials have been carried out to evaluate the efficacy and safety of MLT. However, the standard protocol for MLT was still not available, and studies continued to explore it [4–8]. Besides, even though some papers evaluated MLT’ efficacy, there’s still no data showed how long the IOP-reduction effect could last [9]. This study aimed to illustrate the effects of 360° MLT’s on Chinese patients with glaucoma or ocular hypertension within a longer follow-up period.

## Methods

This was a prospective study conducted at the Second Xiangya Hospital from 2015 to 2019. All the patients were recruited from the Department of Ophthalmology.

The inclusion criteria were as follows: (1) patients of Asian origin with glaucoma or ocular hypertension (OHT); (2) age older than 18 years. The exclusion criteria were as follows: (1) received glaucoma surgery within 3 months; (2) history of trauma; (3) subjects who were unable to be followed-up. Slit-lamp examination and gonioscopy were performed, IOP was measured using Goldmann applanation, and visual field (VF) was also tested using the Humphrey Field Analyzer perimeter. Open-angle glaucoma (OAG) was defined as the open angle on gonioscopy (Shaffer grading > 2 in all quadrants) in the presence of a progressive increase in C/D ratio or VF defects. Patients with higher IOP, typically 21 mm Hg, but no VF or retinal nerve fibre layer (RNFL) defects were classified as OHT [10]. Two eyes diagnosed with primary angle-closure glaucoma were included after LPI treatment, with Shaffer grading ≥ 2 in 360°. Four eyes received filtering surgery before but IOP was not well controlled were also included. The patients were explained about the choices they had and the possibilities of adding other treatments. Informed consents were obtained from all participants.

Prior to MLT, topical anaesthesia (proparacaine hydrochloride 0.5%) was applied to the treated eye 5 min apart. All patients received a single session of MLT by the same surgeon using a Diode True-Yellow 577-nm Laser with MicroPulse technology (IQ 577 TM Laser System, Iridex Corporation, 1212 Terra Bella Avenue, Mountain View, CA, USA).

Patients went home immediately after the procedure. Nonsteroidal anti-inflammatory drugs (Pranoprofen, 0.1%) was applied four times a day for 1 week. BCVA and IOP measurement were routinely carried out on day 0, day 1 and in week 1, month 1, month 3, month 6, month 18, and month 36. Anti-glaucoma drugs were titrated up or down when the IOP was higher or lower than the targeted one. When multiple drugs were needed, the combination medications were applied to simplify the drug regimen. If the IOP or optic nerve damage was progressed at maximal drug regimen during more than two visit intervals, the patients received additional therapies, such as another laser treatment or filtering surgery.

### Ethical Approval

This study adhered to the tenets of the Declaration of Helsinki. Informed patient consent and approval from the Institutional Review Board of The Second Xiangya Hospital were obtained.

### Statistical Analysis

Statistical analysis was performed using IBM SPSS Statistics (IBM Corp.) and GraphPad Prism 7 (GraphPad Software, Inc.). Repeated-measures analysis of variance (ANOVA) was used to compare IOP values at different time points with Dunnett's multiple-comparisons test for between-groups analysis. A Kaplan–Meier analysis was performed to tell the time to failure after MLT. For all statistical analyses performed, differences were considered statistically significant at *P* < 0.05.

## Results

A total of 39 eyes from 25 patients were enrolled in this study. The patient demographics are shown in Table 1.

**Table 1:** Patient Demographics

Baseline characteristics	*N* = 39
Laterality (right/ left)	24/15
Gender (men/ women)	10/15
Age (years) (mean ± SD)	41.47 ± 14.39
Type of glaucoma	
Ocular hypertension	15
POAG	15
PACG	2
Glaucomatocyclitic crisis	2
Corticosteroid glaucoma	2
Glaucoma after silicone oil removal	1
Juvenile glaucoma	2
Number of medications	
0	17
1	14
2	7
> 2	1
Type of anti-glaucoma medications	
Beta-blocker	11
Prostaglandin agonists	9
Carbonic anhydrase inhibitor	4
Alpha agonist	8
Surgery before MLT	
LASIK	3
LPI	2
Silicone oil tamponade and removal	1
Scleral buckle	1
Trabeculectomy	2
Ex-PRESS shunt surgery	2
Mean C/D ratio (mean ± SD)	0.63 ± 0.22
Visual Field	
Visual filed index (%) (mean ± SD)	84 ± 25
Mean deviation (dB) (mean ± SD)	-5.44 ± 6.69
Pattern standard deviation (dB) (mean ± SD)	5.92 ± 7.51

MLT was completed in all eyes with the laser settings shown in Table 2

**Table 2:** Laser Settings

Laser wavelength	577 nm
Contact lens	MLT lens
Spot size	300 μm
Power	1000 mW
Location of treatment	Pigmented trabecular meshwork
Degree of treatment	360 degree (130 shots)
Treatment duration	300 ms
Duty cycle	15% duty, 85% rest

No complication occurred during or after MLT. The average follow-up time was 35 ± 1.53 months (range 34.45 to 35.55 months). The mean IOP and number of medications at different time intervals following MLT for all patients were listed in Table 3.

**Table 3:** Changes in IOP and Number of Medications at Different Time Intervals following MLT

	Pre-ML *N* = 39	1 Day *N* = 39	1 Week *N* = 37	1 Month *N* = 37	3 Months *N* = 37	6 Months *N* = 36	18 Months *N* = 34	36 Months *N* = 32
IOP (mm Hg ± SD)	21.13 ± 7.75	18.79 ± 7.17	20.61 ± 4.81	19.45 ± 4.70	18.43 ± 4.28	17.52 ± 4.25	18.01 ± 4.20	18.56 ± 5.66
Range	(18.62–23.64)	(16.47–21.12)	(19.01–22.22)	(17.89–21.02)	(17.01–19.86)	(16.09–18.96)	(16.55–19.48)	(16.52–20.60)
Number of medications	0.82 ± 0.91	0.46 ± 0.77	0.30 ± 0.74	0.57 ± 0.88	0.59 ± 0.86	0.69 ± 0.62	0.62 ± 0.65	0.69 ± 0.64

**Fig. 1:**
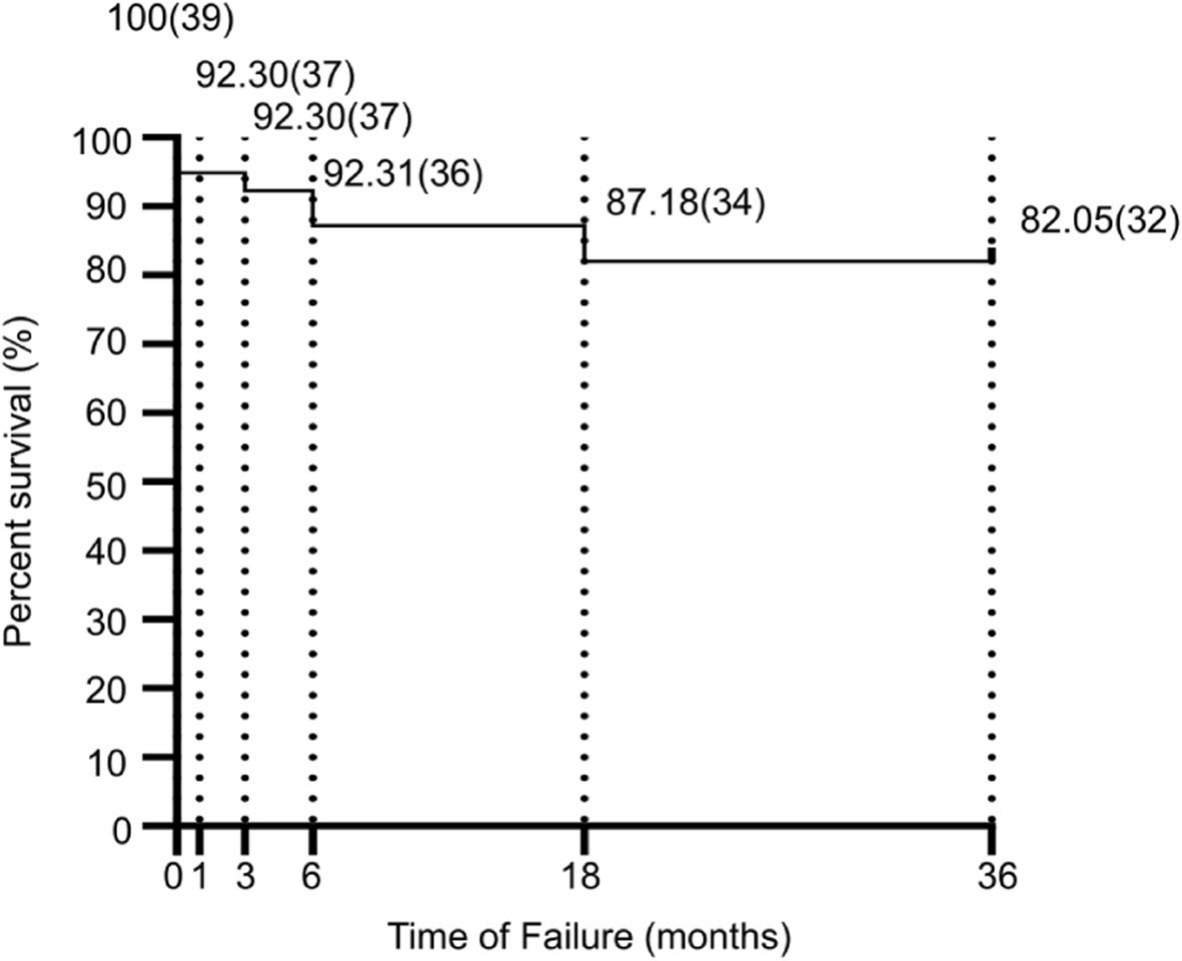
Kaplan-Meiersurvival Curve Analysis Plotting the Cumulative Probability of No Failure after MLT. Percent survival (number of eyes) are presented

During the follow-up period, seven eyes received additional laser or surgical treatment. These procedures included selective laser trabeculoplasty (SLT) in 4 eyes, shunt implantation in 2 eyes, and trabeculectomy in 1 eye. A survival curve analysis is presented in Fig. 1, in which mortality is defined as the presence of progressive IOP elevation or optic nerve damage, due to which the patients received further laser or surgical treatment.

**Fig. 2:**
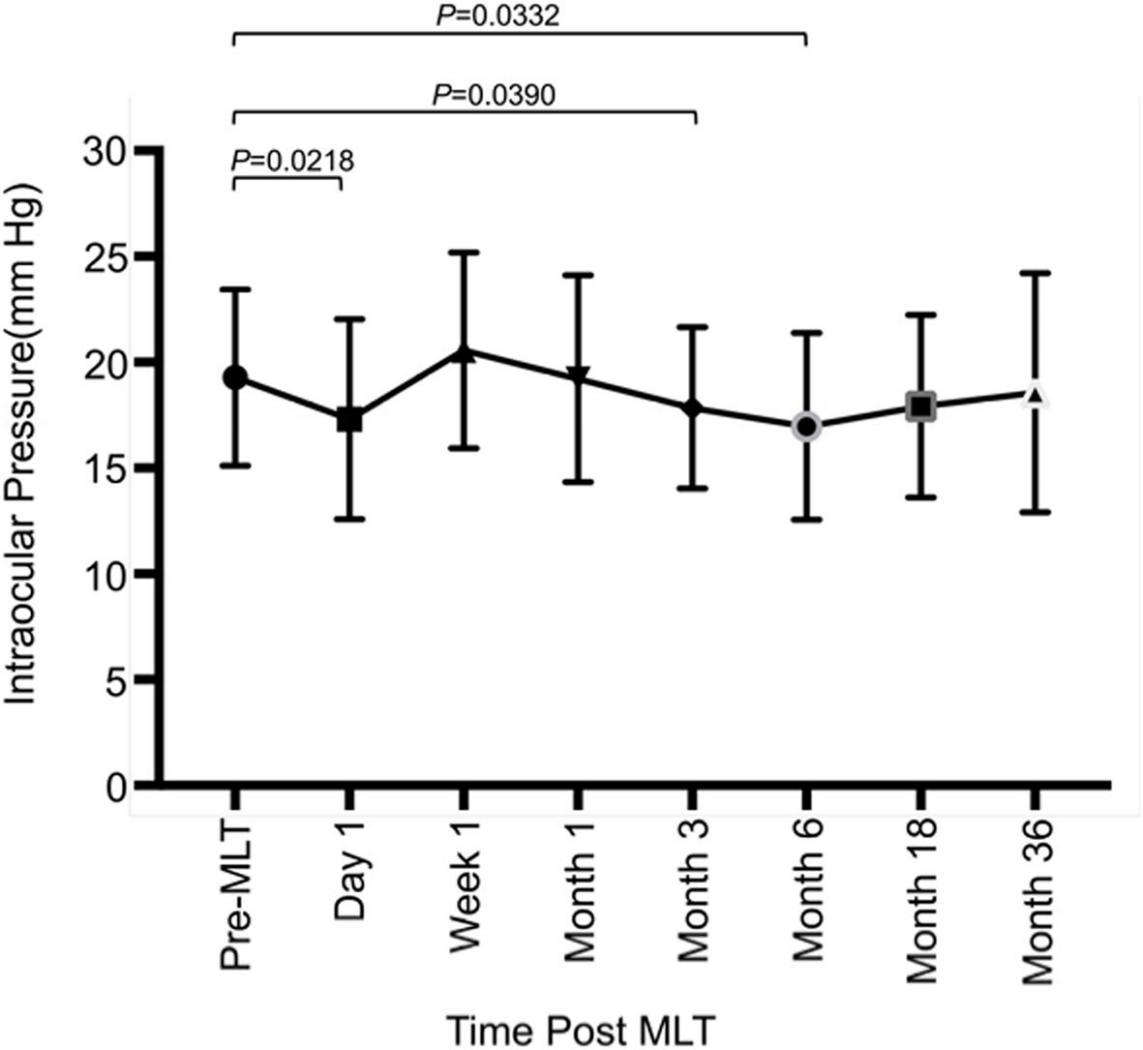
Changes in IOP following MLT with SD bars above and below the Mean. MLT = Micro-Pulse laser trabeculoplasty

Only patients without MLT treatment failure, who could be evaluated from the beginning to the end of the 35-month observation period, were analyzed. The Kolmogorov–Smirnov test was performed (*P* > 0.05), which demonstrated that the data was suitable for repeated-measures ANOVA analysis. After 1 day, 3 months, 6 months, and 36 months of MLT, more than 20% patients exhibited a more than 20% IOP reduction, while the mean IOP was significantly reduced at 1 day (q = 3.12, *P* = 0.0218, DF = 31), 3 months (q = 2.876, *P* = 0.039, DF = 31), and 6 months (q = 2.945, *P* = 0.0332, DF = 31) visit compared with the pre-MLT level (Table 4, Fig. 2, *P* < 0.05). On day 1(q = 5.23, *P* = 0.0001, DF = 31) and in week 1 (q = 4.836, *P* = 0.0002, DF = 31), the number of medications is significantly reduced (Table 4, *P* < 0.05). Besides, at the end of the study, the number of eyes needed prostaglandin increased from 8 to 17. However, no significant difference was observed in the number of anti-glaucoma medications between the pre-operative visit and the last visit.

**Table 4:** Changes in IOP and Number of Medications at Different Time Intervals following MLT for Patients without MLT Treatment Failure

	Pre-ML *N* = 32	1 Day *N* = 32	1 Week *N* = 32	1 Month *N* = 32	3 Months *N* = 32	6 Months *N* = 32	18 Months *N* = 32	36 Months *N* = 32
IOP (mm Hg ± SD)	19.28 ± 4.17	17.31 ± 4.72	20.55 ± 4.63	19.22 ± 4.88	17.84 ± 3.81	16.97 ± 4.42	17.92 ± 4.31	18.56 ± 5.66
Range	(17.78–20.78)	(15.61–19.01)	(18.89–22.22)	(17.46–20.98)	(16.47–19.22)	(15.38–18.56)	(16.37–19.48)	(16.52–20.60)
Percent reduction (%)	–	10.21	-6.60	0.32	7.46	12.0	7.05	3.73
*P*	–	*0.0218*	0.3130	0.9999	*0.0390*	*0.0332*	0.0986	0.9248
Number of medications:	0.78 ± 0.79	0.31 ± 0.47	0.25 ± 0.51	0.47 ± 0.67	0.53 ± 0.62	0.59 ± 0.67	0.59 ± 0.67	0.69 ± 0.64
*P*	–	0.0001	0.0002	0.1115	0.4033	0.6642	0.7397	0.9652
Number of patients with prostaglandin:	8	1	1	6	9	12	12	17
≥ 20% IOP reduction (%)	-	25.0	0	6.25	21.88	28.13	15.63	21.88

**Fig. 3:**
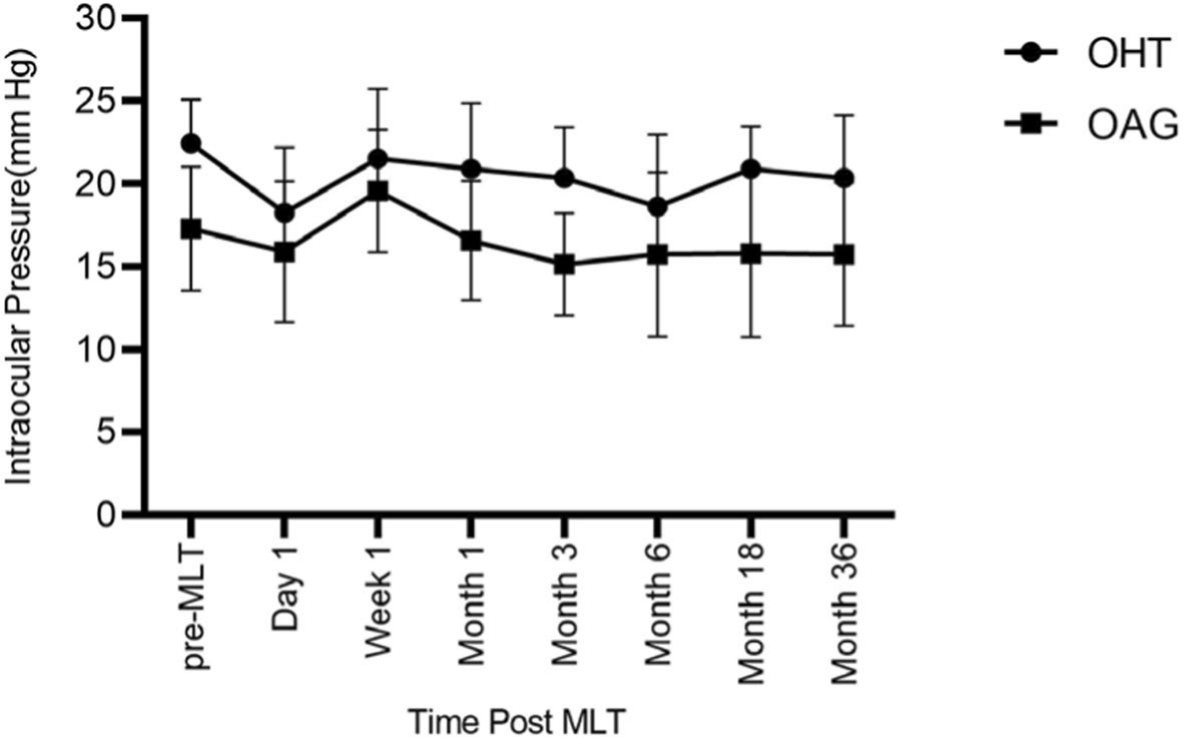
Changes in IOP following MLT for patients with OHT or OAG with SD bars above and below the mean

In addition, patients with OHT or OAG could be comparatively analyzed during the observation period, as seen in Table 5 and Fig. 3. After 1 day, 3 months, 6 months, 18 months, and 36 months of MLT, more than 20% patients with OAG exhibited a more than 20% IOP reduction, while more than 20% patients with OHT exhibited a more than 20% IOP reduction after 1 day, 6 months, and 36 months of MLT. The OHT patients had a higher average starting IOP (22.45 mmHg) than the OAG group (17.31 mmHg), approaching significance (*P* = 0.0009). After 1 day of MLT, there was no significant difference in IOP between the above groups, while the IOP of OAG patients was significantly lower than the OHT patients at 1 month (*P* = 0.0124), 3 months (*P* = 0.0004), 18 months (*P* = 0.0061) and 36 months (*P* = 0.0119). Moreover, the number of medications between groups showed no significant difference at follow-up intervals (Table 6).

**Table 5:** Changes in IOP at Different Time Intervals following MLT for Patients with OHT or OAG

		**Pre-ML**	**1 Day**	**1 Week**	**1 Month**	**3 Months**	**6 Months**	**18 Months**	**36 Months**
OHT (*N* = 11)	IOP (mmHg ± SD)	22.45 ± 2.62	18.27 ± 3.95	21.55 ± 4.18	20.91 ± 4.00	20.36 ± 3.08	18.64 ± 4.34	20.91 ± 2.59	20.36 ± 3.76
	Range	(18.00–26.00)	(13.00–26.00)	(16.00–28.00)	(16.00–26.00)	(14.00–24.00)	(11.00–24.00)	(17.00–25.00)	(14.00–27.00)
	≥ 20% IOP reduction (%)	–	36.36	0	9.09	18.18	36.36	9.09	27.27
OAG (*N* = 13)	IOP (mm Hg ± SD)	17.31 ± 3.73	15.92 ± 4.27	19.59 ± 3.70	16.58 ± 3.61	15.15 ± 3.08	15.77 ± 4.97	15.81 ± 5.04	15.77 ± 4.34
	Range	(9.00–21.00)	(5.00–20.00)	(15.00–27.70)	(10.00–22.00)	(10.00–19.00)	(11.00–28.00)	(11.00–28.00)	(11.00–28.00)
	≥ 20% IOP reduction (%)	–	23.08	0	7.69	38.46	38.46	30.77	30.77
	*P*	0.0009	0.1787	0.2373	0.0124	0.0004	0.1502	0.0061	0.0119

**Table 6:** Changes in Number of Medications at Different Time Intervals following MLT for Patients with OHT or OAG

		Pre-ML	1 Day	1 Week	1 Month	3 Months	6 Months	18 Months	36 Months
Mean number of medications ± SD	OHT (*N* = 11)	0.36 ± 0.50	0.18 ± 0.40	0.18 ± 0.40	0.36 ± 0.50	0.36 ± 0.50	0.36 ± 0.50	0.55 ± 0.52	0.55 ± 0.52
	OAG (*N* = 13)	0.54 ± 0.52	0.23 ± 0.44	0.15 ± 0.38	0.54 ± 0.78	0.77 ± 0.733	0.84 ± 0.69	0.58 ± 0.51	0.69 ± 0.48
	*P*	0.4139	0.7804	0.8623	0.5287	0.1329	0.0673	0.8627	0.4808

## Discussion

As universally acknowledged, argon laser trabeculoplasty (ALT) exerts thermal damage to the treated TM, causing the shrinkage of collagen fibres, stretching and widening the adjacent areas and allow more outflow [11]. Compared to ALT, SLT’s energy selectively absorbed by pigmented trabecular cells and makes no damage to adjacent structures. Both ALT and SLT provided reliable IOP-lowering effect, but ALT caused obvious damage to TM histologically [11, 12]. Several clinical trials claimed that SLT was an effective way to control IOP in multiple types of glaucoma [13–17]. However, a wide range of complications, such as redness, photophobia, peripheral anterior synechiae, and cystoid macular oedema were still observed in patients with SLT [18].

Unlike the former laser technologies, MLT is based on a micropulse laser with a 15% duty cycle, which allows the temperature to return to the baseline. Under this condition, both TM and Schlemm’s canal could escape from cellular damage, scarring, and morphological changes [19].

Previous research demonstrated that MLT (810 nm) was less effective in reducing IOP compared with ALT on a 3-month visit [20]. A retrospective review was conducted by Rantala, in which they evaluated 180° MLT (810 nm, 180°) on patients with OAG with a minimum follow-up period of 6 months, and found that MLT was a safe but not effective-enough treatment for glaucoma [4]. Lee investigated the use of 360° MLT (577 nm) for treating patients with OAG. By the end of the 6-month visit, 72.9% of patients were successfully treated [5]. Abramowitz carried out a prospective randomized clinical trial, comparing 360° MLT (577 nm) with 360° SLT. He showed that MLT was as effective as SLT within a 52-week follow-up period [21]. Valera-Cornejo conducted MLT (532 nm, 360°) on patients with OAG and found that it reduced IOP in a pretty short period [6]. All of the above indicated that both the range and the wavelength of laser applied to patients may affect the results. In the present study, IOP was averagely reduced by 3.73%, and only 21.88% of the patients achieved a more than 20% reduction in IOP by the end of the 35-month visit, despite adding a topical therapy of prostaglandins to 9 more eyes (from 8 to 17). Furthermore, compared with the OHT group, the IOP-reducing effect of MLT was more pronounced in the OAG group. This result was not as encouraging as the previous findings. Recently, several studies claimed that MLT is a safe and effective way to attenuate IOP, while the observation period were relatively short [8, 22–24].

The present study had several limitations. Initially, this was a small-sample study. Patients’ characterizations, such as the optic nerve condition, might have affected the results. Seven eyes with a C/D ratio range from 0.9 to1.0 were enrolled, four of them underwent trabeculectomy, demonstrating a poor prognosis. The reason why we recruit them is because several anti-glaucoma drugs had been prescribed but not sufficient to lower the IOP in these patients. To reduce the glaucoma medications and slow down the progression of optic nerve damage, we decided to perform MLT to these 4 eyes. Secondly, given the relatively long follow-up period, it was hard for patients to completely following doctors’ instructions. Especially, those who still have a relatively good visual acuity, might not apply drugs as routinely as required, leading to an under-estimated IOP-lowering effect. Lastly, this inhomogeneous cohort whose patients enrolled suffered from many different types of glaucoma might have influenced the results enormously.

To our knowledge, this research was one of the longest explorations of the effect of MLT on patients with glaucoma and OHT, claiming that a single session of MLT could significantly reduce IOP in a pretty short period. Given the repeatability of MLT, it could be used as an IOP-lowering treatment.

## Conclusion

MLT is a newly glaucoma treatment based on a micropulse laser with a 15% duty cycle, which could reduce IOP in patients with glaucoma or ocular hypertension for a short period, but its lowering efficiency was limited up to 6 months of the follow-up period.

## Data Availability

The datasets used and analyzed during the current study are available from the corresponding author on reasonable request.

## Abbreviations

ANOVA: Repeated-measures analysis of variance
BCVA: Best-corrected visual acuity
C/D ratio: Vertical cup-to-disc ratio
IOP: Intraocular pressure
LTP: Laser trabeculoplasty Laser trabeculoplasty
MLT: Micropulse laser trabecular plasty
OH: Ocular hypertension
RNFL: Retinal nerve fibre layer
SLT: Selective laser trabeculoplasty
TM: Trabecular meshwork
VF: Visual field
